# Patscanui: an intuitive web interface for searching patterns in DNA and protein data

**DOI:** 10.1093/nar/gky321

**Published:** 2018-05-02

**Authors:** Kai Blin, Wolfgang Wohlleben, Tilmann Weber

**Affiliations:** 1 The Novo Nordisk Foundation Center for Biosustainability, Technical University of Denmark, Kgs. Lyngby, Denmark; 2 Interfaculty Institute for Microbiology and Infection Medicine, Eberhard Karls University of Tübingen, Tübingen, Germany

## Abstract

Patterns in biological sequences frequently signify interesting features in the underlying molecule. Many tools exist to search for well-known patterns. Less support is available for exploratory analysis, where no well-defined patterns are known yet. PatScanUI (https://patscan.secondarymetabolites.org/) provides a highly interactive web interface to the powerful generic pattern search tool PatScan. The complex PatScan-patterns are created in a drag-and-drop aware interface allowing researchers to do rapid prototyping of the often complicated patterns useful to identifying features of interest.

## INTRODUCTION

Many interesting biological features can be identified based on characteristic patterns in DNA and protein sequences. Bioinformatics tools and databases make use of such sequence patterns to address specific research questions, from identifying potential sgRNA sequences in CRISPR applications (such as CRISPy-web (1)) or identifying tandem repeats (2) to the PROSITE Database (3). However, these tools usually are restricted to their very specific use-case and mostly use pre-defined static patterns to search for. If new motifs are identified in research papers or using motif discovery tools like MEME (4) or HOMER (5), it often takes significant time until they find their way into dedicated software tools. Being able to efficiently search for arbitrary sequence patterns directly allows researchers to bridge this technological gap.

In 1997, Dsouza *et al*. published the generic pattern search tool PatScan (6), allowing to search for DNA and protein sequence patterns using an expressive pattern language: It is possible to specify patterns that match complex structures such as repeats, hairpins, stem loops and pseudoknots. Weight matrices can be used as patterns, and patterns may also contain ambiguity codes for nucleotides. Patterns can be repeated, with or without mismatches, insertions, and deletions, and complemented.

PatScan is available from the SEED servers website (http://blog.theseed.org/servers/2010/07/scan-for-matches.html). Unfortunately, PatScan is not easily accessible to researchers without scripting background as it is a command-line only tool that also requires learning the complex pattern language. There is no built-in help function, constructing more complex patterns can quickly turn unwieldy, and there is no syntax checking to help finding typos in patterns.

In order to solve the usability challenges and make flexible pattern searching more available to the biology community, we have developed an interactive web-based interface, PatScanUI. Our web server supports all pattern types used in the command-line utility while ensuring that all elements of the pattern are valid and provides extensive help and tutorial sections. While creating patterns, parts of the pattern can be rearranged using simple drag & drop operation.

## DESIGN AND IMPLEMENTATION

PatScanUI consists of two components. The browser-based web client is implemented in JavaScript and handles creating and validating the pattern set users want to run. The web client uses Knockout.js (http://knockoutjs.com/) to link the underlying data model to the user interface. The data model verifies that only valid patterns can be created and submitted to the server. On the server side, the Python-based Flask framework (http://flask.pocoo.org/) handles file uploads, receiving search pattern sets and running the PatScan command line tool.

**Figure 1.**
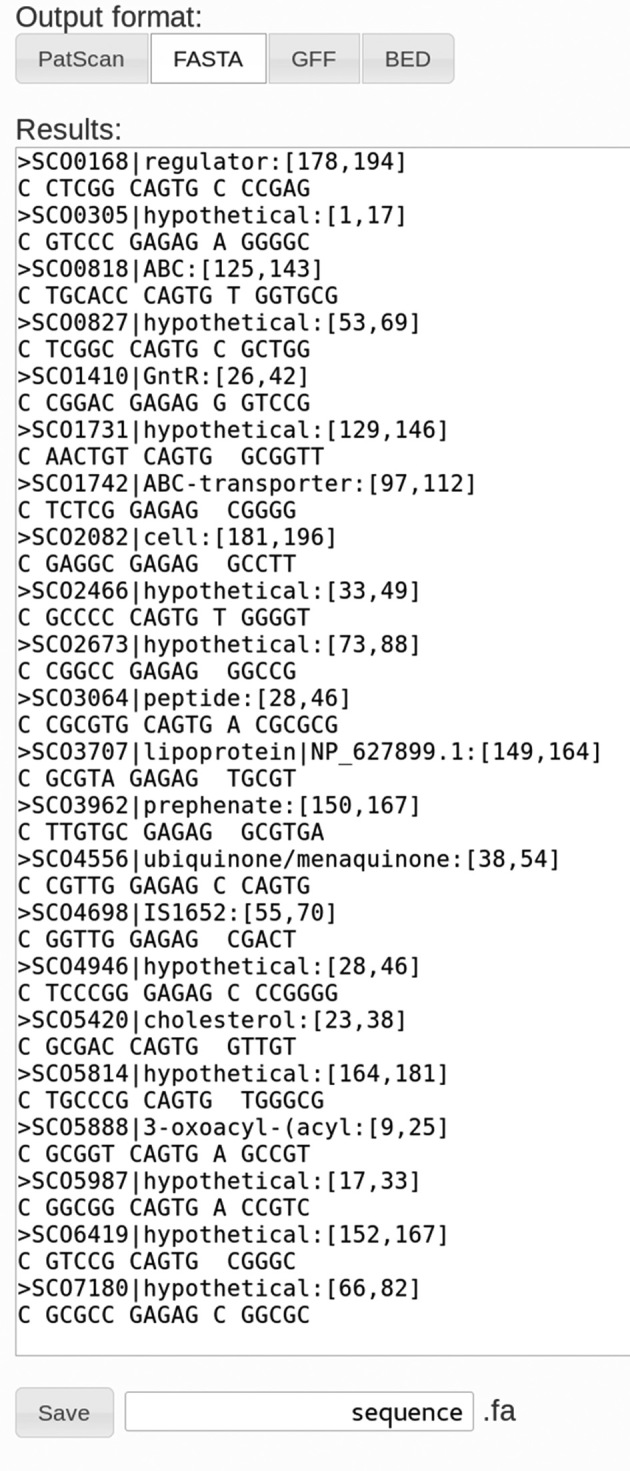
PatScan output displayed in FASTA format. Different output formats can be selected using the buttons on top. The ‘Save’ button below the text field allows saving the output to a file in the selected format.

PatScanUI accepts FASTA-formatted input files containing one or more sequences and provides output in four different formats. The PatScan command line tool returns matches in FASTA format, and also has a further processing script that returns a more compact representation. In addition to these two formats, PatScanUI also can output the matches in GFF3 and BED format. Outputs are shown in a text box for quick iterations while developing the pattern, and can also be downloaded in any of the four formats (Figure 1).

## FEATURES AND APPLICATIONS

PatScanUI supports all pattern types supported by the command line tool. Available patterns depend on the input molecule, as some pattern types are available for DNA/protein sequences only. String patterns take a string of bases or amino acids that should be identified in the input sequences. For DNA input, the IUPAC ambiguity codes may be used as well. Range patterns match a length of arbitrary nucleotides or amino acids and are usually used as spaces between more specific patterns. Complement patterns are exclusive to DNA inputs and match the reverse complement of a previously defined pattern. Repeat patterns match a previously matched pattern again. Alternative patterns take any two patterns and require just one of them to match. Length limit patterns allow adding a length constraint to previous matches and are usually utilized with multiple range patterns. Weight patterns present a hybrid of position specific scoring and probability matrices for DNA sequences. The probability of encountering a specific base in a particular position in percent are used to sum up a total match score, the weight. The weight pattern also takes a minimum score threshold, allowing to filter out matches that are not good enough. On protein inputs, the ‘any of’ and ‘not any of’ allow to specify a list of amino acids that need to be present or absent at a particular position. In order to support unusual base-pairings in e.g. RNA sequences, alternative complementation rules can be defined to specify which base can pair with which. String, complement, and repeat patterns also support matching with variations, so it is possible to specify how many mutations, insertions or deletions are acceptable for the pattern to still match.

With PatScanUI, all these pattern types—or combinations thereof—can be interactively explored and assembled. In order to better explain the various pattern types supported by PatScan, we present an exemplary biological application. Starting out with a relatively simple pattern, we will keep adding more pattern types to capture more of the biological nuances and demonstrate how PatScanUI can be useful for quick exploratory analysis of a sequence. For even more detail on how to run the analysis, see the tutorial on the PatScanUI website.

### Example: finding iron-response elements in bacteria

Besides its catalytic function in the TCA cycle, the aconitase enzyme has an important regulatory function in both Eukaryotes and Prokaryotes. When active as a regulator, aconitase binds specific structural motifs in mRNA, so-called iron-response elements (IREs). In Eukaryotes, aconitase is highly sensitive to iron deprivation and oxidative stress (7,8). In bacteria, the role of aconitase seems to be similar (9), though the recognition sequence is less preserved (10). Aconitase recognises the RNA stem loop structure of the IRE consisting of a C bulge, a stem of six nucleotides, and a loop of five or six nucleotides. In Eukaryotes, the loop sequence always is CAGUG, in Bacteria, more variations to both stem and loop are possible. In this example, we will build up a set of patterns to identify putative bacterial IREs in the bacterium *Streptomyces coelicolor*. The 5′ UTR sequences used for this example and a step-by-step guide are provided on the PatScanUI tutorial page (https://patscan.secondarymetabolites.org/tutorial).

The basic IRE pattern is CN_1_N_2_N_3_N_4_N_5_N_6_CAGU GN'_6_N'_5_N'_4_N'_3_N'_2_N'_1_, where N_*x*_ is any nucleotide, and N'_*x*_ is its complement (7). An initial attempt at finding such a pattern using PatScan would use a number of string patterns and a complement pattern. Just using a string pattern with CNNNNNNCAGUGNNNNNN would fail to ensure the second set of Ns would be the reverse complement of the first, so instead we need to break the pattern up into three pieces:

One for the C base, one for the first half of the stem consisting of six Ns, and one for the loop section consisting of the CAGUG part. A complement pattern for the second half of the stem then captures the complete IRE.

On the example input of *S. coelicolor* 5′ UTR regions, this pattern does not give any results. The reason for this is that the IRE motif in bacteria is more variable (9). Notably, the stem region does not need to have six perfect matches. Using the ‘variations’ option on the complement pattern to allow one mismatch relaxes the requirement enough to find hits. The loop region in IREs can contain an additional base after the CAGUG sequence (7), this can be supported by adding a range pattern for a range from 0 to 1.

Another variation that is common on bacterial IREs is that the stem of the stem loop structure is only five bases long, not six. Replacing the string pattern of Ns with another range pattern allows this.

Not only the stem region but also the loop region can have variations in bacteria. Notably, the loop sequence GAGAG has also been reported to be functional (10). To support searching for CAGUG or GAGAG at the same time, we can use the alternative pattern. It is basically a container that can hold any two other patterns, one of which has to match.

**Figure 2.**
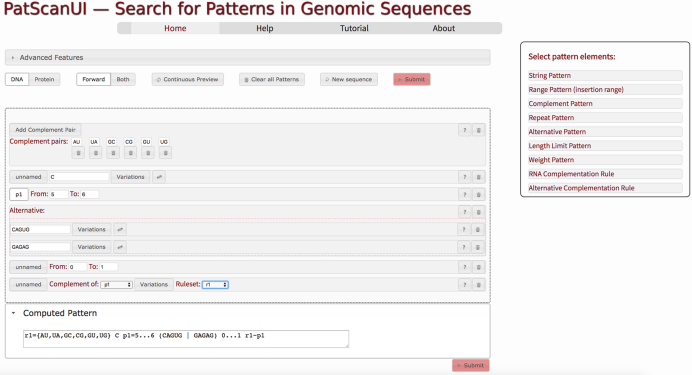
UI-driven design of complex PatScan patterns. The individual parts of the pattern—in this example the search for IRE elements—can be interactively assembled by dragging the respective ‘pattern element’ into the working area. PatScanUI then generates the PatScan pattern, which is used to search the submitted sequence by pressing the ‘Submit’ button.

By default, PatScan uses the regular DNA complementation rules to determine which bases can be in a reverse complement. IREs are RNA sequences, so additional GU/UG pairs are possible. To support this, you can create custom alternative complementation rules (Figure 2).

If we wanted to explore further alternatives to the loop pattern sequence, we could nest multiple ‘alternative patterns’ to create a deeper logical branching. That approach does get a bit unwieldy, though. A better option is to instead use a weight pattern.

## CONCLUSIONS

PatScanUI provides a user-friendly interface to design and develop complex search patterns for various applications based on the powerful PatScan pattern-matching engine. Due to the possibility to interactively edit and optimize the patterns, the tool is well suited to rapidly prototype pattern-matching strategies, without requiring scripting or command-line tools.

## DATA AVAILABILITY

PatScanUI is available from https://patscan.secondarymetabolites.org/. This website is free and open to all users and there is no login requirement. Source code is available under the OSI-approved AGPL license from https://github.com/kblin/patscanui/.
